# Social bonding drives vocal exchanges in Bonobos

**DOI:** 10.1038/s41598-018-36024-9

**Published:** 2019-01-24

**Authors:** Florence Levréro, Sonia Touitou, Julia Frédet, Baptiste Nairaud, Jean-Pascal Guéry, Alban Lemasson

**Affiliations:** 1 Université de Lyon/Saint-Etienne, Equipe Neuro-Ethologie Sensorielle, Neuro-PSI, CNRS UMR 9197 Saint-Etienne, France; 2 La Vallée des Singes, 86700 Romagne, France; 3 Université de Rennes, EthoS “Ethologie Animale et Humaine,” UMR 6552 –CNRS-Université de Caen Normandie, Station Biologique, 35380 Paimpont, France

## Abstract

The origin of human speech is still a hotly debated topic in science. Evidence of socially-guided acoustic flexibility and proto-conversational rules has been found in several monkey species, but is lacking in social and cooperative great apes. Here we investigated spontaneous vocal interactions within a peaceful context in captive bonobos to reveal that vocal interactions obey temporally and social rules. Dyadic vocal interactions were characterized by call overlap avoidance and short inter-call intervals. Bonobos preferentially responded to conspecifics with whom they maintained close bonds. We also found that vocal sharing rate (production rate of shared acoustic variants within each given dyad) was mostly explained by the age difference of callers, as other individual characteristics (sex, kinship) and social parameters (affinity in spatial proximity and in vocal interactions) were not. Our results show that great apes spontaneously display primitive conversation rules guided by social bonds. The demonstration that such coordinated vocal interactions are shared between monkeys, apes and humans fills a significant gap in our knowledge of vocal communication within the primate phylogeny and highlights the universal feature of social influence in vocal interactions.

## Introduction

The evolutionary origins of language and speech remains a fundamental question in science. In particular, whether clues to the origins of speech are present in nonhuman primate communication remains a hotly debated topic^1–4^. Despite the diversity of social cultures and languages in humans, universal features in conversations are found across all languages, such as the avoidance of overlapping and a minimum gap between turns^5–8^. Orderly vocal exchanges (antiphony between two or more animals or duets within male-female pairs^9^) have been found across the primate order: from lemurs^10^, to New World monkeys^11–15^, Old World monkeys^16^ and lesser apes^17,18^. Vocal turn-taking appears to be associated with social life and cooperation capacities^1,2,5,9,19–21^. It is thought to maintain and reinforce social bonds between individuals (e.g. in non-human primates^10,22^), enable the extraction of information in the absence of overlap (e.g.^23^ but see^24^) and reduce stress as in the case of social grooming^25^.

Vocal exchange is “a characteristic communication style in which a sender produces a vocalization to address a receiver, and the receiver emits a call in response within a brief interval” (cited from^26^). Vocal exchange patterns are influenced by social factors in non-human primates. ‘Interlocutors’ are not randomly selected, and preference is given to elders^11,27–29^, social allies^12,14,22^ or dominant individuals^30,31^. The attention of the audience also influences vocal outputs leading to persistence (repetition of calls) and elaboration (changes in the acoustic structure of calls) in situations where no response has been received^32,33^. Shared primitive forms of vocal turn-taking within non-human primate species might suggest an ancient evolutionary origin^1,34^. Surprisingly, however, studies based on great apes are scarce and controversial. No evidence of spontaneous vocal coordinated exchanges has been found in wild chimpanzees^35^, who display complex social interactions and cooperative abilities^36^. Indeed, Arcadi^35^ found that chimpanzees do not “respond” to the majority of calls they heard (within 5 sec), and that instead, bonded males tend to chorus together, matching each-other’s pant hoots^37,38^. Nevertheless, a recent study in great apes found for the first time that captive gorillas display some rule-governed call exchanges^31^. Relying on our current knowledge, vocal turn-taking is thus reported across phylogenetically distant species (monkeys and more generally in some social mammals such as African elephants^39^, bottlenose dolphins^40^, bats^41,42^, naked mole-rats^43^) but with some apparent discontinuities among great apes. More investigations among great ape species, our closest, highly social, relatives, are thus necessary in order to ascertain if vocal-turn taking behavior is as a result of convergent evolution (analogies as adaptations to similar social requirements) or is shared ancestry (homologies which are inheritance behaviours)^34^.

Social influences on the acoustic patterns of calls can also be assessed by examining the rate of vocal sharing, defined as the production rate of vocal ‘variants’ between individuals at a given time^26^. The study of vocal sharing in nonhuman primates provides insight into vocal flexibility, and particularly into the flexibility of call production and call use. In several species, it has been shown that a given individual can produce several acoustic variants (i.e. stereotypical patterns of frequency modulation) of the same affiliative call type at a given stage of its life, with some variants being potentially shared with preferred group members e.g.^26,44^. Vocal sharing between affiliative or kin partners has been identified in mouse lemurs^45^, pygmy marmosets^46^, Wied’s black-tufted-ear marmoset^47^, Campbell’s monkeys^26,48^, siamang gibbons^49,50^ and recently in captive chimpanzees^51^. However, what adaptive benefits are conferred by vocal sharing remains an open question. The most parsimonious explanation is that it functions to reinforce social bonding, facilitating navigation (mate identification and reunion, joint territory defense) and aiding social integration (gibbons^49,50^, marmosets^52,53^, chimpanzees^38,51,54^ and humans^55,56^).

In this study, we attempted to identify features that are already known to characterize both monkey call exchanges and human conversations in a great ape species, the bonobo (*Pan paniscus*). Bonobos live in dense social network with a fission-fusion social system^57^ and present high cognitive and cooperative abilities e.g.^58^. As with chimpanzees, the vocal mode of communication plays an important role in bonobos due to their forest habitat. Their vocal repertoire is graded^59,60^, meaning that call sub-structures are extremely variable. Surprisingly, to the best of our knowledge, neither the temporal organization of vocal interactions nor the extent of vocal sharing, have previously been investigated in bonobos. Here, to plug this gap, we examined the temporal organization of vocal interactions and the social factors that influence vocal sharing in a captive group of bonobos. We expected to observe some level of vocal turn-taking in specific contexts and to identify social factors that control the patterns of vocal exchanges. More specifically, we predicted more frequent vocal interactions and a higher rate of vocal exchanges between preferred social partners.

## Results

### Occurrence of ‘vocal responses’

**Figure 1:**
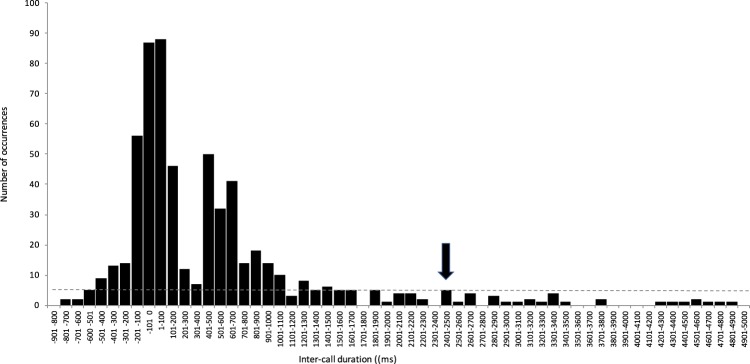
Distribution of inter-call durations between different consecutive callers recorded from 70 independent focal samples of 10 minutes each (n = 779 inter-call durations). Grey dotted line: baseline of the occurrence of inter-call durations. Black arrow: threshold indicating the maximum response delay of vocal exchanges (i.e. when the number of occurrences starts to be above the baseline level).

We studied whether call utterances within the group occurred randomly or whether they were temporally organized with a given call being followed by a so-called ‘vocal response’^37^. In peaceful contexts (see Methods), we found that calls often elicited a vocal response from other group members. Vocal responses were, however, not systematic and three calling patterns were observed: overlapped calling, successive calling and isolated calling (Fig. 1). Overlapped calling consisted in calls emitted simultaneously by different individuals (i.e. inter-call durations were negative, meaning that the onset of the call “response” started before the offset of the first call and consequently the two calls partially overlapped) (Fig. 1). Successive calling, sometimes referred to antiphony, consisted of a temporal synchronization with no overlap of the call utterances between two consecutive callers (i.e. inter-call durations were positive and short, meaning that there was a short silence between the two consecutive calls) (Fig. 1; see also a typical sequence of vocal exchanges in the Supporting Audio File). Isolated calling consisted of calls emitted independently from the call utterances of other group members (i.e. inter-call durations were above 2500 ms, which has been defined as the baseline threshold) (see Fig. 1).

**Figure 2:**
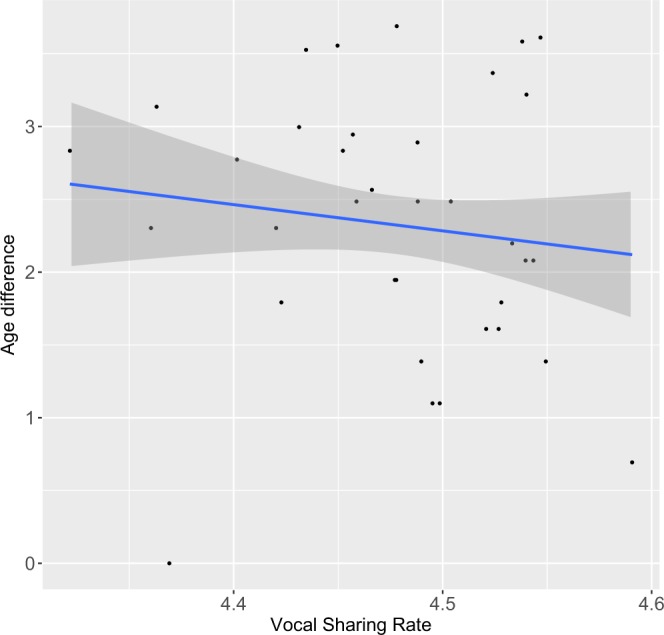
Effect of age difference on vocal sharing rate. ‘Vocal sharing’ was calculated for each dyad as the average of individual similarities in ‘variant’ rates of each acoustic pattern. Predictors were log-transformed. Solid line: linear regression of the estimated marginal means, grey shaded area: confidence interval.

In total we recorded 779 call intervals within 70 ten-minute sequences. We recorded 48.8% of successive calling (designated as vocal exchanges hereafter; 385/779 call intervals), 26.4% of isolated calls (206/779) and 24.1% overlapped calling (188/779). Vocal exchanges thus followed two basic temporal rules: (1) relatively rare call overlap, since 67.2% of inter-call durations were positive (*n* = *385/573*; Fig. 1), and (2) short response delay (<2500 ms). Interestingly, Fig. 1 shows a bimodal distribution with a first peak including both overlapped calls (between −200 ms and 0 ms) and positive and very short call intervals (between 1 ms and 200 ms). The second peak of the bimodal distribution (Fig. 1) shows that positive inter-call durations were usually above 400 ms (60.3% of positive inter-call durations n = 232/385), indicating that individuals typically leave a gap of a bit more than an average call duration (247 ms ± 179 ms, this study) before responding, thus preventing overlap.

Group members did not equally participate in vocal exchanges (n = 310 calls extracted from a sub-sample composed of 192 vocal exchanges (see Methods and Table S1); *χ²* = *146*.*9*, *df* = *8*, *P* < *0*.*001*).

Lastly, when we examined all vocal sequences (i.e. not only dyadic vocal exchanges but all vocal sequences composed of several calls spaced by less than 2500 ms each; n = 118), we observed that vocal sequences typically involved two or three callers (78.3% and 18.1% of the vocal sequences respectively; mean 2.2 ± 0.5 individuals, maximum number of individuals: 4), with an average of 3.7 ± 2.1 calls in vocal interactions.

### Classification of calls

The 310 exchanged calls were pre-classified into six acoustically distinct ‘variants’, based on the shape of fundamental frequency modulation pattern through visual inspections of spectrograms (see Methods and Fig. S1). Most of the exchanged calls (83%) corresponded to “peep yelps” and “peeps” according to bonobo repertoire in literature (see Methods and Fig. S1 for more details and comparisons with call types classically described in bonobo literature). A PCA followed by a DFA analysis, based on six acoustic parameters measured in the temporal and spectral domains (call duration, start frequency, maximum frequency, end frequency, ascending slope, and descending slope; see details in Methods) validated this classification. The averaged correct classification of the different ‘variants’ was 91.6% (min-max: 60–100%), largely higher than a correct classification by chance (one chance out of six: 16.7%). All the acoustic parameters significantly participated in discriminating the six ‘variants’ (*Wilks*’ *lambda* = *0*.*034*, *F*_*(approx*.*30*, *1198)*_ = *53*.*019*, *P* < 0.001, see Fig. S1 for more details).

### Production rate of vocal ‘variants’

During vocal exchanges, the six identified ‘variants’ were not produced in similar proportions (*χ²* = *171*.*4*, *df* = *5*, *P* < *0*.*001*) with the variants A, B and C being the most frequent (34.8%; 25.5% and 24.5% respectively, Table S1).

The production rate of the different ‘variants’ differed between individuals (χ² = 67.86, df = 40, P = 0.004). The most often recorded ‘variant A’ (108/310 calls) represented 12.5% to 66.7% of the calls in individual repertoires. Of the six defined ‘variants’, variants A, B and D were produced by all the studied individuals (n = 9), variant C by eight of the individuals and variants E and F by less than half of the individuals. In order to identify the factors explaining these individual differences, we compared hereunder the vocal sharing rate, which is the production rate of vocal ‘variants’ between individuals calculating a vocal similarity score per pair of individuals (see Methods for a complete definition and calculation detail).

### Socio-demographic determinants of vocal sharing

We assessed the influence of individual characteristics (age, sex, kinship) and social (affinity) factors of interacting partners on vocal sharing rate. Social affinity between individuals was defined by dyadic spatial proximities (see Methods). We found that social affinity differed among bonobos. Indeed, most of bonobos did not spend their time randomly in proximity to each group member (for seven ind.: *30*.*5* < *χ²* < *187*.*9*, *df* = *7*, *P* < *0*.*001*; for one ind: *χ²* = *21*.*1*, *df* = *7*, *P* < *0*.*01*; for one ind: χ*²* = *1*.*6*, *df* = *7*, *P* = *0*.*97*). We found that only age difference between callers significantly affected vocal sharing rate (i.e. differences in call rates for each given dyad, see Methods; *n* = 72; GLM: *χ²* = *8*.*19*, *df* = *1*, *P* = *0*.*004*; Fig. 2, Table 1). The greater the age difference between two individuals, the less likely they are to share acoustic ‘variants’. The other tested fixed factors (social affinity rate, vocal affinity rate (i.e. dyadic vocal response rate, see Methods for detailed definition), sex composition of vocal dyads and kinship) were not significant predictors of the vocal sharing rate (see Table 1). Interestingly, all the variants could induce a vocal ‘response’ (Fisher’s Exact Test *P* = *0*.*95*, Table S1) and the matching of ‘variants’ within vocal exchanges was not systematic. Indeed, we found that only 36.5% of vocal exchanges (n = 192) were composed of similar ‘variants’ (Table 2). It is worth noting that this rate still remains higher than ‘variant’ matching by chance (16.7%). A similar result was found when only taking into account the first two callers of all the vocal sequences recorded (composed of several calls spaced with a short response delay, see above) (39% of ‘variant’ matching, n = 118 of vocal exchanges between the two first contributors of vocal sequences; see Methods).

**Table 1:** Statistical analysis of the effect of socio-demographic determinants on vocal sharing rate between two consecutive callers.

Fixed effects	χ²	d.f	P-value
Social Affinity	0.132	1	0.717
Vocal Affinity	1.089	1	0.765
Sex composition of vocal dyads	0.031	1	0.86
Age difference	8.19	1	**0.004**
Kinship	1.313	1	0.252

P-values were obtained independently for each predictor with likelihood-ratio tests comparing the fit of the full model with a reduced model lacking the fixed effect. Significant P-values are given in bold. Generalized Linear mixed model (GLMM) with a Gaussian error structure. Normality of the residuals was attained after applying a log-transformation.

**Table 2:** Matrix representing the percentage of the different ‘variant’ combinations in vocal exchanges (n = 192 pairs of consecutive calls).

Voc 1	Voc 2					
	A	B	C	D	E	F
A (62)	48.4 (30)	17.7	22.6	9.7	1.6	0
B (51)	29.4	37.3 (19)	19.6	9.8	3.9	0
C (50)	22	26	30 (15)	14	4	4
D (19)	42.1	15.8	21.1	21.1 (4)	0	0
E (5)	0	20	20	40	20 (1)	0
F (5)	40	0	20	20	0	20 (1)

On the first left column, “Voc 1” is the ‘variant’ starting a vocal exchange and in bracket is the number of observations where each variant was produced in first position. “Voc 2” is the ‘variant’ given in “response” namely produced within a maximum delay of 2.5 sec (see the body text for the definition of this threshold). ‘Variant’ matching is on the diagonal line and in brackets is the number of vocal exchanges where Voc 2 was the same that Voc 1.

### Socio-demographic determinants of vocal affinity

**Figure 3:**
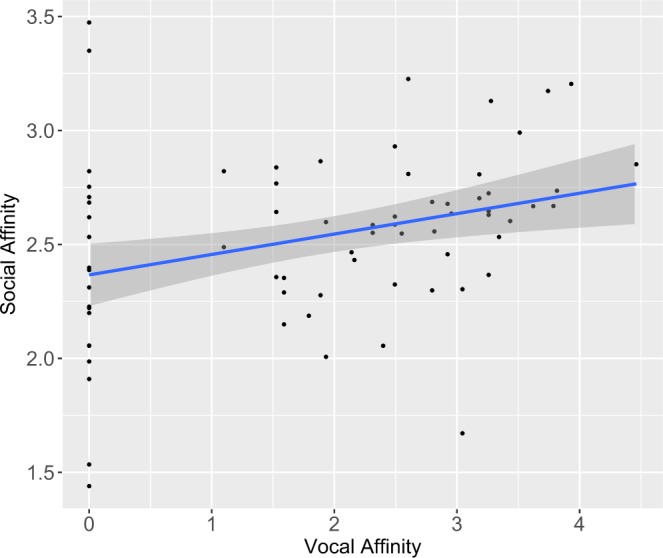
Effect of social affinity on vocal affinity. ‘Social affinity’ was calculated for each dyad as the frequency of occurrences of peaceful spatial proximities and ‘vocal affinity’ as the frequency of their vocal interactions. Predictors were log-transformed. Solid line: linear regression of the estimated marginal means, grey shaded area: confidence interval.

We assessed the influence of individual characteristics (age, sex, kinship) and social (affinity) factors of interacting partners on their amount of vocal interactions. Vocal affinity (the frequency of vocal interactions for each given dyad, see Methods for detailed definition) was only explained by the ‘social affinity’ of individuals (*n* = 72; GLM: *χ²* = *8*.*4*, *df* = *1*, *P* = *0*.*003*; Fig. 3 Table 3) while the sex of callers, age differences and kinships were not significant predictors. Thus the stronger the ‘social affinity’ between two individuals, the more they are preferred vocal partners.

**Table 3:** Statistical analysis of the effect of socio-demographic determinants on vocal affinity between individuals.

Fixed effects	χ²	d.f	P-value
Social Affinity	8.4	1	**0.00375**
Sex composition of vocal dyads	0.009	1	0.923
Age difference	1.795	1	0.180
Kinship	2.764	1	0.096

P-values were obtained independently for each predictor with likelihood-ratio tests comparing the fit of the full model with a reduced model lacking the fixed effect. Significant P-values are given in bold. Generalized Linear mixed model (GLMM) with a Gaussian error structure. Normality of the residuals was attained after applying a log-transformation.

**Figure 4:**
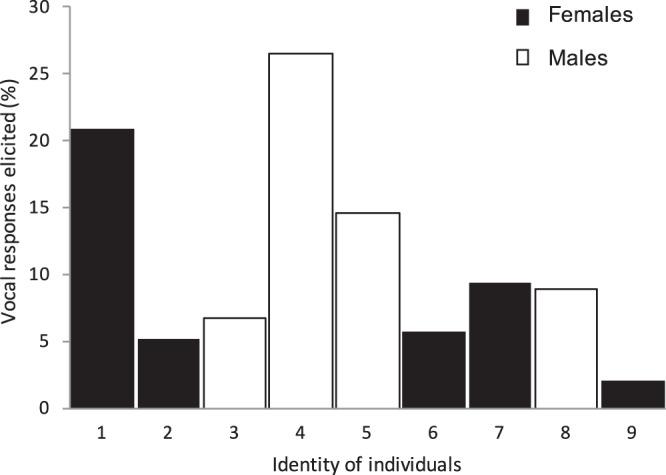
Vocal responses elicited by the different group members. Percentage of times that a call of a given bonobo elicited a vocal response from a conspecific. Individuals are ordered by decreasing age. Individuals 1 and 4 correspond to a mother-son pair.

**Figure 5:**
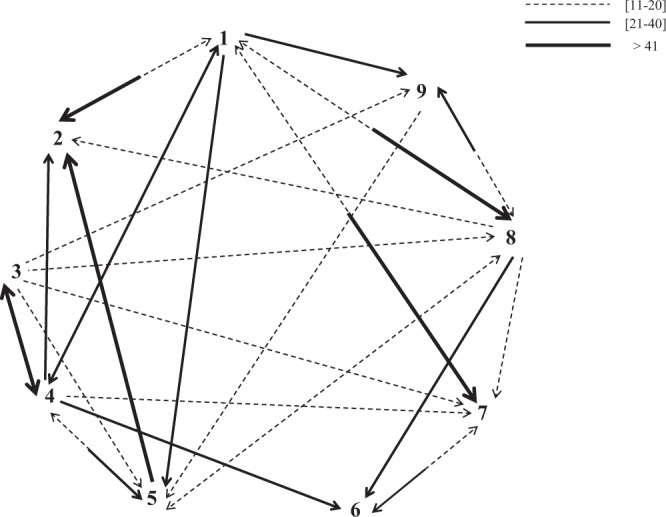
Sociogram showing vocal affinities. The arrows indicate who responds frequently to whom. Only dyadic interactions occurring more often than a threshold score of 10% are drawn here. The thickness of the arrows is related to the frequency of the interaction.

Lastly, we identified preferred exchanging partners amongst individuals, as some bonobos induced more responses from specific group members than others (*χ²* = *89*.*77*, *df* = *8*, *P* < *0*.*001*, Figs 4 and 5). The dominant female (ind. 1) and her adult son (ind. 4) induced the highest numbers of vocal responses.

## Discussion

Evidence of human-like conversations is scarce in great apes. Vocal exchanges have been reported in the context of long-distance communication in chimpanzees^61^ and bonobos^62^, as a means to coordinate their movement between parties. Here we demonstrate for the first time that orderly vocal exchanges in short distance communication exist in bonobos. We show that their vocal exchanges respect basic temporal (i.e. turn-taking, overlap avoidance) and social (i.e. interlocutor selectivity) rules, similar to the rules guiding human conversations. Second, vocal sharing (i.e. typically resulting from a progressive vocal convergence between at least two individuals) was determined by the age of individuals, independently of their social affinity, sex and kinship. Third, the frequency of vocal interactions between interlocutors was only influenced by social bonds.

In the context of spontaneous vocal interactions at short distances and in a peaceful context, we found that bonobos displayed two vocal interaction patterns (overlapped and successive calling). Overlapped calling may function as the well-known chorus in chimpanzees to maintain spatial contact over long distances^63^ (notably to locate and facilitate reunions with other community members, especially males) while successive calling (vocal exchanges hereafter), which until now has only been described in gorillas^31^ among great apes, may have a significant social function similar to the vocal exchanges in monkeys and human conversations. Among nonhuman primates, spontaneous orderly vocal exchanges had only been described in monkey species (e.g. vervets, macaques, marmoset *sp*., squirrel monkeys, Campbell’s monkeys) until we recently identified them in gorillas^31^. In orangutans, an experimental turn-taking setup showed that this ape has the cognitive control of vocal turn-taking^64,65^. Overall, this suggests that spontaneous vocal exchanges may be spread among great ape species and that the lack of evidence is likely as a result of the lack of investigations. The only study conducted in chimpanzees concluded that they produce non-temporally organized call utterances or synchronous choruses^35^. Their social behaviour might be less favourable to vocal exchanges than in bonobos. While bonobos are known for displaying pacifist behaviour and for their use of sexual behaviour in conflict appeasement, chimpanzees were found to resort more to force to deal with social conflicts^58^. The absence of vocal turn-taking in chimpanzees, however, remains puzzling considering their complex social interactions and cooperative abilities. This finding in chimpanzees may be explained by the study conditions, which greatly differed from the bonobo and gorilla studies, and should perhaps be considered a preliminary result. First, the chimpanzees’ study did not focus on peaceful contexts, which are more susceptible to induce organized vocal exchanges. Second, contrary to other ape studies, it took place in the wild where soft calls may have been undetected, as acknowledged by the author. Lastly callers were all adult male chimpanzees. Further research is thus necessary to investigate potential spontaneous cooperative vocal communication with vocal turn-taking behavior in captive chimpanzees. More generally, tracking the evolutionary pathway of proto-conversation within the primate lineage will require more comparative studies between socially-diverse species to discuss a potential convergent evolution or an ancestral origin of vocal cooperation.

Vocal sharing in bonobos had not been previously been investigated. Among their graded vocal system^59,60^, we identified distinctive acoustic ‘variants’ and found differences in their production rate among individuals. Focusing on these ‘variants’ in the context of vocal exchanges, only age difference explained the vocal sharing rate in bonobos. Vocal sharing was not influenced by their social affinities or by other individual characteristics (sex and kinship). This finding differs from the studies on Campbell’s monkeys^26,48,66^, chimpanzees^37,54^ and humans^67^ for which social affinity predicts vocal sharing. It also differs from Japanese macaques for which vocal sharing is predicted by dominance ranks^68^. The question of the functions of vocal sharing in vocal exchanges is still open. Sharing acoustic ‘variants’ may function as a ‘vocal social badge’ to advertise bonding within a group. This is the case with most monkey species e.g.^26,53,66^ and also with chimpanzees^37^. Social systems may greatly influence the importance of signaling specific social bonds, such as hierarchical bonds in despotic societies^68^, male-female affinities in monogamous groups^53^ and female-female affinities in uni-male/multi-female groups^48^. To the best of our knowledge, no studies had previously reported that age influences vocal sharing. We found in bonobos that the greater the age difference between two callers, the less likely that they shared acoustic ‘variants’. We can thus hypothesize that juvenile bonobos display a ‘youngster vocal badge’ since they often play with peers (but not exclusively see^69^), which may favor the development of social bonds during their long immaturity period. It is also interesting to note that an effect of age has been found on call exchange rate in gorillas^31^. The closer two gorillas are in age, the more likely they are to exchange grunts. These observations might thus suggest that age proximity plays an important role in great apes that are characterized by a long period of development and their longevity. At this stage, we cannot conclusively rule out alternative hypotheses, although these appear less plausible: (i) Vocal sharing might indeed result due to the phenomenon of maturation. Although a link between age and morphological changes in vocal apparatus has been shown in numerous species^70^, it seems unlikely in bonobos since all age classes produced the recorded ‘variants’ in our focused group; (ii) Vocal sharing could be context-specific but in the present study all vocal exchanges were recorded in a relative standardized context (peaceful context); (iii) Vocal features could depend on the internal state of callers (review in mammals including primates^71^); (iv) Young individuals might share more similar emotions with their peers than with other age classes, which might explain the correlation found between age and vocal sharing rate in bonobos.

Interestingly, bonobos do not match the ‘variants’ during their vocal exchanges contrary to what is suggested for some monkey species (Diana monkeys^72^; Japanese macaques^73^) and in most of birds who display song matching^46^. This is however similar to what was described in Campbell’s monkeys where individuals who share the same acoustic variants do not systematically respond to each other by matching their calls^26^.

Lastly, we found that vocal interactions between individuals were affected by their social affinities. Indeed, bonobos did not randomly respond to any group members but preferentially to some specific conspecifics with whom they maintain close bonds. This finding is consistent with the evolutionary function of call exchanges or chorusing which is often linked to social bonding (e.g. many pair-living bird species^74^, primates species: ring-tailed lemurs^75^, squirrel monkeys^14,76,77^, spider monkeys^78^, rhesus macaques^79^, siamangs^17^, gorillas^80^ and chimpanzees^37^). Interestingly, we observed an unbalanced vocal response rate between individuals (individual B systematically calls after individual A but the contrary is not true) that suggests that vocal exchanges may reflect an active search of building bonds. Hierarchical ranks may also influence the patterns of vocal exchanges, but more groups are however needed in order to test this effect. It is widely considered that socially-ruled communicative behaviour shared by humans and nonhuman primates may have been a crucial step in the coevolution of language and social life e.g.^1,2,20^. In line with this, Dunbar^25^ described nonhuman primate call exchanges as ‘vocal grooming’. When social groups became too large and too complex, limiting the possibility to interact physically with a lot of members, vocal exchanges, and then later language, could have become a way to maintain close bonds at distance.

In conclusion, this study conducted on a single captive group of bonobos shows that great apes, like monkeys, apply some simple conversational turn-taking rules and only social bonds determine the frequency of these vocal exchanges. Moreover, the sharing of vocal patterns appears to be essentially influenced by the age of bonobos, independent of their social bonds. These findings fill an important gap regarding the knowledge of vocal patterns along the primate phylogeny. Whether the main role of vocal exchanges is to reinforce social bonds between vocal interlocutors, advertise the existing ones or to convey more informative signals remains to be determined by conducting further playback experiments. We suggest that further comparative work, and the consideration of multiple sensory modalities in investigations of turn-taking behavior^81^ are now required to identify the key factors that have driven the emergence of the different components of human language.

## Methods

### Ethics Statement

All methods were carried out in accordance with the relevant French national guidelines and regulations, under the authorization no. C42-218-0901-38 SV 09 delivered by the «Direction Départementale de la Protection des Populations» committee, Préfecture du Rhône. This study only involves behavioural observations and spontaneous vocal recordings of animals in their natural social group and in their usual environment. No experimental protocols have been conducted in this study.

### Study site and animals

A captive group of nine bonobos composed of four males (8 to 20 years old) and five females (5 to 44 years old) was observed at the zoological Park of La Vallée des Singes (Romagne, France). Wild bonobos live in fission-fusion social system^82,83^ and their community size greatly varies according to the studied field sites^82,84,85^. The studied group composition reflects natural mixed subgroups observed in the wild. No studied individuals have been rescued or experienced bad treatment. The group was initially composed of five individuals (ind. #1, 3, 4, 5, and 9; Figs 4 and 5) who have lived together for at least four years and one year later, joined by four additional individuals (April-May 2011) before our data collection started. The study group was composed of the following pairs: three mother/offspring, one father/offspring, one half-brother, one nephew and one second-cousin. They lived in a large enriched building of 900 m² with access to a one-hectare wooded island depending on the meteorological conditions. Bonobos were fed eight times per day with fresh fruits and vegetables. Water was available *ad libitum*.

### Data collection

Bonobos were monitored about six hours per day during 31 days from March to May 2012. Observations consisted in alternating focal group and scan sampling. Focal groups lasted 10 minutes each during which all vocal utterances were recorded (Sennheiser MKH70 ultra-directional microphone, Marantz PMD670 recorder 44.1 kHz sample rate/16 bit resolution, Sennheiser HD 25-1 II headphones) with the identity of the callers given in a handheld digital voice recorder (Yamaha Pocketrak2G). Between two given focal group samples, scan sampling was scored every 15 minutes to identify the closest neighbour of each group member and record their respective activity using the aforementioned voice recorder. We focused on vocal interactions produced within peaceful contexts such as resting and foraging periods, and peaceful social interactions like grooming sessions. Calls recorded in agonistic interactions (e.g. conflicts characterized by chasing, hitting and biting behaviours) or in response of external disturbance (e.g. arrival of caregivers) were thus excluded from the subsequent analyses. Indeed, turn-taking and vocalization spacing require calmness, control and attention toward the others while excitation or conflicts may lead to more overlap (e.g. in humans^7,86^). The distance between individuals involved in these vocal exchanges were between less than 1 meter and about 8 meters.

### Occurrence of ‘vocal responses’

The group vocalized in 55% of the focal group samples performed (70/127 ten-minute sequences). To describe the temporal organization of call production, we studied the distribution of all the inter-call durations from distinct identified consecutive callers^29^. When there were more than two exchanged calls we focused on each of the consecutive calls: for example, in calls exchanged between the individuals ABC, the vocal dyads AB and BC were studied; between ABA, the vocal dyads AB and BA were studied (as in^31^). We are aware that such divisions in dyads are a debated question, so for the study of the matching of ‘variants’ we also report the analysis with only the first two callers (i.e. in a sequence between ABC, we only considered AB). We measured on spectrograms the time intervals between two consecutive calls from the offset of a call to the onset of the next one (using PRAAT software version 5.3.56). A negative time interval meant that the two calls overlapped. In total, we computed 779 inter-call durations, which were used to assess the time window referring to, what could later be named a ‘vocal response’, defining the consecutive pairs of exchanged calls without overlap. To compare the proportion of calls eliciting a vocal response according to the identity of the emitters, we calculated the percentage for each individual of his/her total number of calls produced during the focal group samples that were followed by a consecutive caller within 2500 ms (i.e. response delay threshold, see Fig. 1).

### Classification of calls

310 exchanged calls were isolated from 192 vocal exchanges, a sub-sample of our data taken in chronological order of the recordings. These 310 calls were pre-classified into acoustic ‘variants’ based on the shape of fundamental frequency modulation pattern through visual inspections of spectrograms (see Fig. S1), as is classically done with birds^87^, monkeys^66^ and cetaceans^88,89^. The bonobo vocal repertoire is described as highly graded, namely that there are no clear boundaries in the acoustic structure of the different call types (see the spectrograms in^59,60^). In the present study, most of exchanged calls were soft and short as expected in peaceful context and mostly corresponded to “peep yelps” and “peeps” according to the bonobo repertoire in the literature. Peep yelps and peeps are both described in literature as presenting “very variable shapes”^59,90^. Peep yelps and peeps can have a flat, ascending, descending or double frequency modulation. Peeps are also characterized by their short duration^60,91^ (<100 ms). Since we focused on call production within only one general peaceful context, we looked for more subtle call classification based on their frequency modulation patterns. We thus obtained 6 ‘variants’ (Fig. S2) from the visual inspection of spectrograms (with PRAAT software version 5.3.56, available from www.praat.org): **Variant A**: no frequency modulation, varied duration (n = 108 calls classified as variant A). **Variant B**: ascending frequency with an optional plateau at the end (start frequency < end frequency), varied duration (n = 79). **Variant C:** frequency modulation with an ascending and a descending slope (start frequency = end frequency), varied duration (n = 76). **Variant D (**opposite to variant 2): descending frequency modulation with an optional plateau at the beginning (start frequency > end frequency), varied duration (n = 33). **Variant E:** frequency modulation with an ascending and a descending slope (start frequency > end frequency), short duration (n = 9). **Variant F:** strong frequency modulation with two ascending and two descending slopes, long duration (n = 5). Sixty one percent of calls were classified for accuracy by a second observer (FL) and tested using the Cohen’s Kappa coefficient to ensure inter-observer reliability. We obtained a “very good” level of agreement (κ = 0.85).

For general information, we could classify 61.3% of the calls produced during vocal exchanges (N = 310 calls) in call types according to the bonobo repertoire^59^ and found 51% of peep yelps, 32% of peeps, 6% of soft barks and less than 3% of barks, bark screams, whistles, whine whistles, pout moans, yelps, whistle barks and grunts). Bermejo & Omedes^59^ described various call types (e.g. peep yelp, peep, soft bark, whistle) produced in peaceful contexts (foraging, grooming, resting, play incitation, play sequences). Our classification in ‘variants’ from A to F thus distinguishes the acoustic variations within call types. A call type can be found in different ‘variants’ (each of our ‘variants’ includes at least two distinct call types).

### Acoustic measurements

Six classical acoustic parameters had been manually measured on the fundamental frequency with PRAAT software using the following settings: analysis window length 0.05 s, dynamic range 70 dB; spectrogram view range 0–8 kHz. In temporal and frequency domains we measured: call duration (d), start frequency (Fstart), maximum frequency (i.e. highest pitched frequency, Fmax), end frequency (Fend), ascending slope ((Fmax-Fstart)/duration) and descending slope ((Fmax-Fend)/duration). A Principal Component Analysis (PCA) followed by a Discriminant Function Analysis (DFA) was performed to test the relevance of our variants pre-classification^16^.

### Socio-demographic determinants

For each possible dyad of bonobos (bidirectional data: N = 36) we calculated the ‘age difference’ in years and the ‘coefficient of relatedness’. We then calculated for each ‘variant’ a dyadic ‘vocal sharing’ score (n = 36) which was the differences in call rates of the two individuals forming a given dyad. Individual call rates were the number of times that each variant was uttered divided by the total number of calls produced by one individual, then multiplied by 100. For each dyad, the differences of call rate between all ‘variants’ were then averaged; we thus obtained a score of vocal dissimilarity that we subtracted from 100 to get a more intuitive score (i.e. the higher the score, the higher the acoustic similarity between two individuals). We also calculated, based on the identification of the closest neighbour of each individual from 487 scan samples, a dyadic ‘social bonding’ score quoted thereafter ‘social affinity’ (unidirectional data: N = 72) relying on the frequency of occurrence of peaceful spatial proximities. Individuals were considered in peaceful spatial proximity when they interacted positively (e.g. grooming) or rested or foraged at 5 meters or less from each other (see also^92^). The score for dyad AB was calculated according to the formula: number of scans where B is the closest neighbour (and is in peaceful spatial proximity) of A * 100/total number of scans where A had been observed. Last the dyadic vocal response rate, quoted ‘vocal affinity’ thereafter (n = 72) was calculated for each dyad. For the dyad AB for instance, we recorded the percentage of times A got a vocal response from B out of the total A calls and *vice versa* (i.e. the higher the score, the higher the vocal affinity between two individuals). Unilateral dyads were considered here since reciprocity for vocal affinity was not observed within dyads, namely individual A can elicit more vocal response rate from individual B than B from A.

### Statistical analyses

#### Studied group

Because the studied group resulted of the fusion of two former groups (composed of five and four individuals respectively) one year before our study (see above in Methods), we checked that the nine individuals truly formed a united group in terms of social and vocal behaviour. When necessary we performed generalized linear mixed models (GLM), which account for the repeated variables of identity (i.e. the same individual was involved in several vocal or social dyads). First no differences were found in the production rate of the six ‘variants’ regarding the past membership of individuals (for each variant: Mann-Whitney tests: *z* ranged from *0 to 1*.*715* and *P* from *0*.*09 to 1*). Second no social preferences, defined by dyadic spatial proximities (see definition above in Methods) have been highlighted between the individuals who initially belonged to the same subgroup (*n* = 72; GLM: *χ²* = *0*.*55*, *df* = *1*, *P* = *0*.*456*). Lastly, vocal affinity (i.e. dyadic vocal response rate, see above in Methods) between exchanging callers was not influenced by the past membership of individuals (*n* = 72; GLM: *χ²* = *1*.*3*, *df* = *1*, *P* = *0*.*254*; see also Fig. 5). These results showed that the nine individuals indeed form a cohesive group whatever their past membership and can be considered as belonging to a united social group at the time of the study.

#### Socio-demographic determinants of vocal sharing and vocal affinity

We performed GLM (accounting for the repeated variables of identity, namely the same individual was involved in several vocal exchanges) to investigate the socio-demographic factors that influence vocal sharing on the one hand, and on the other hand the vocal affinity (function lmer in lme4 package of R software).

First we tested the effects of social affinity, vocal affinity and individual characteristics (age, sex and kinship) on vocal sharing rate (fixed factors: social affinity, vocal affinity, sex composition of vocal dyads, age difference and kinship; random effects: the identity of the first caller and the identity of the second caller). Second we tested the effects of social affinity and individual characteristics (age, sex and kinship) on vocal affinity (fixed factors: social affinity, vocal sharing, sex composition within vocal dyads, age difference and kinship; random effects: the identity of the first caller and the identity of the second caller). In this model, vocal sharing rate and social affinity*vocal sharing have been excluded from the tested fixed factors since the previous model (see above) revealed that vocal affinity was not a significant predictor of the vocal sharing.

For all analyses, the variables of interests were centered and normalized (i.e. transformed into z-scores) to insure correct weighting since our parameters had different units and then log-transformed to meet the homoscedasticity assumption. We used a GLM with a Gaussian error structure since the normality of the residuals was attained after data transformation. Collinearity was checked for all fixed factors using variance of inflation factor (function vif in car R package). For each predictor we obtained a very low vif (<1.45 for all predictor for vocal sharing analysis, and <1,24 for vocal analysis) indicating that the used predictors were not correlated. The P-values were obtained with likelihood-ratio tests comparing the fit of the full model with a reduced model lacking the fixed effect.

All the statistical tests were performed using R (version 3.4.0) software with a significance level set at α = 0.05.

## Electronic supplementary material

Supplementary Information

Audio file
