# Supplementary material for: Social bonding drives vocal exchanges in Bonobos
Source: Sci Rep. 2019 Jan 24;9:711. doi: 10.1038/s41598-018-36024-9 (PMC6346008; doi:10.1038/s41598-018-36024-9)
Supplement: Supplementary file 1 — Supplementary Information [file 41598_2018_36024_MOESM1_ESM.pdf]

Social bonding drives vocal exchanges in Bonobos

Florence Levréro*[1], Sonia Touitou[1], Julia Frédet[1], Baptiste Nairaud[1], Jean-Pascal Guéry[2] and Alban Lemasson[3]

[1]Université de Lyon/Saint-Etienne, Equipe Neuro-Ethologie Sensorielle, Neuro-PSI, CNRS UMR 9197, Saint-Etienne, France

[2]La Vallée des Singes, 86700 Romagne, France

[3]Université de Rennes, EthoS "Ethologie Animale et Humaine," UMR 6552 –CNRS- Université Caen Normandie, Station Biologique, 35380 Paimpont, France

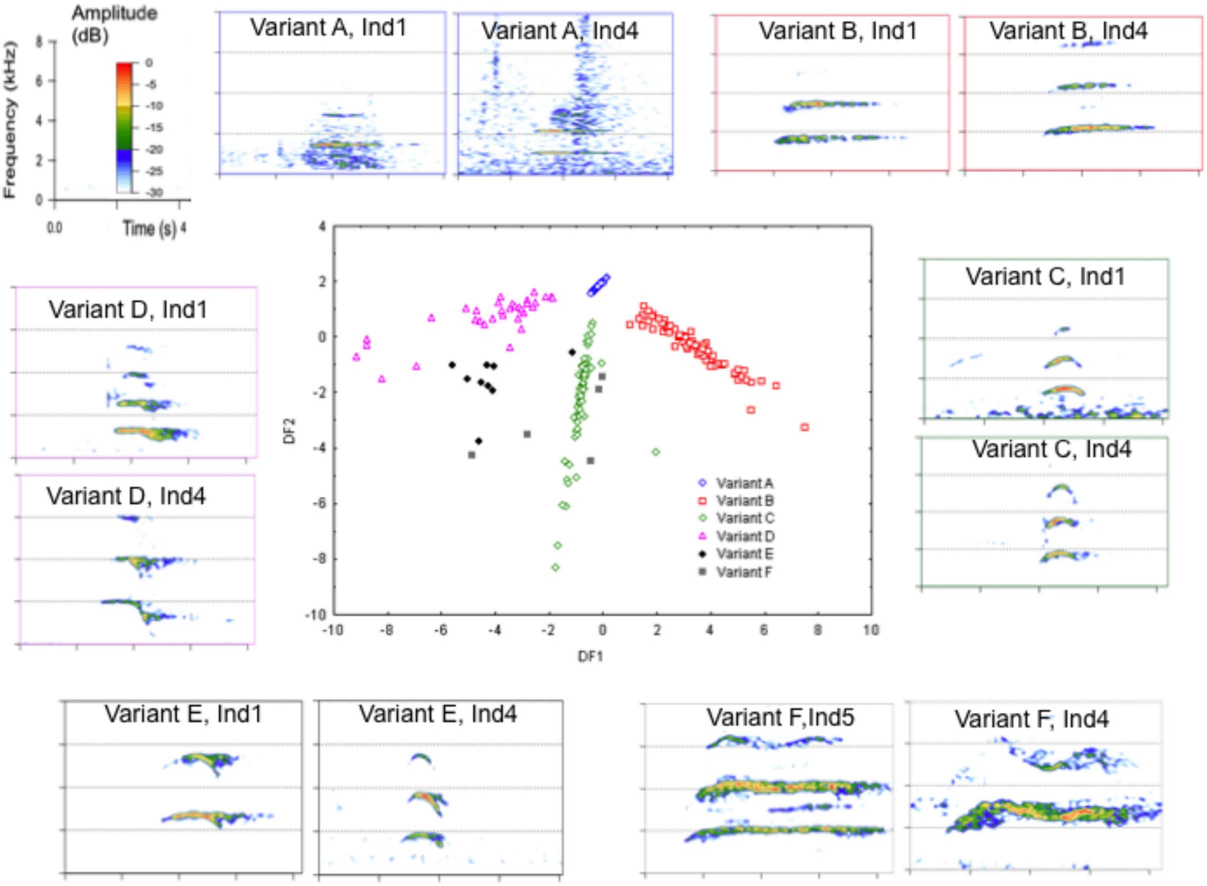

Figure S1. Discriminant analysis ran with the six acoustic parameters measured on 310 calls. Variants (A to F) were previously pre-classified by visual inspection of spectrograms (see Methods for acoustic features justifying this pre-classification). The calls of nine individuals are represented according to the two main functions of the analysis (the first two discriminant functions represented 65.5%, and 32.8% of the total variance respectively). Each pre-classified call variant is represented by a symbol and each point corresponds to one analyzed call. End frequency, Ascending slope and start frequency were the main acoustic parameters responsible for the classification of the variants in the first discriminant function (30.3%, 22.8% and 21.8% respectively). The second function mainly relied on maximum frequency and call duration parameters (30.3% and 23.6% respectively). Spectrograms on the side illustrate the different call variants with two individual callers for each variant. (Spectrograms were plotted using Seewave R package[1]).

Table S1. Comparisons of 'variants' in first position in vocal exchanges.

Variant	Total No. of variants in first position in vocal exchanges	Total No. of recorded variants
**A**	**62**	**108**
**B**	**51**	**79**
**C**	**50**	**76**
**D**	**19**	**33**
**E**	**5**	**9**
**F**	**5**	**5**
**Total**	**192**	**310**

Supplementary Audio file 1: Four vocal exchanges composed of seven calls produced by two individuals.

References

[1]Sueur, J., Aubin, T. & Simonis, C. Seewave: a free modular tool for sound analysis and synthesis. *Bioacoustics* **18**, 213–226, (2008).
